# Ultrastructure of the Epithelium of the Vaginal Complex During the Proestrus and Luteal Phases of the Estrous Cycle in the White‐Eared Opossum (*Didelphis albiventris*)

**DOI:** 10.1111/ahe.70160

**Published:** 2026-07-29

**Authors:** Bruno Cesar Schimming, Tais Harumi de Castro Sasahara, Selma Maria Michelin Matheus

**Affiliations:** ^1^ School of Veterinary Medicine and Animal Science, Graduate Program in Wild Animals São Paulo State University (UNESP) Botucatu Brazil; ^2^ Department of Anatomy, Institute of Biosciences of Botucatu São Paulo State University (UNESP) Botucatu Brazil

**Keywords:** estrous cycle, marsupial, opossum, vagina, wildlife

## Abstract

The white‐eared opossum ( *Didelphis albiventris* ) is a Neotropical opportunistic marsupial widely distributed in the Brazilian territory, occupying both open and forest formations. The anatomy of the marsupial female genital tract consists of two ovaries, oviducts, double uterus, two lateral vaginae, vaginal sinus and a urogenital sinus. The two lateral vaginae, vaginal sinus and urogenital sinus constitute the called vaginal complex that is the most variable part of the anatomy of the marsupial female tract. Thus, this study aimed to investigate the ultrastructure of the vaginal complex in the proestrus and luteal phases in the five females of *D. albiventris* . The vaginal surface is thrown into numerous longitudinal folds separated by furrows. The same morphological architecture was observed in the lining epithelium of the cranial, middle and caudal portions of the lateral vaginae. The lining epithelium of the lateral vaginae presented morphological changes according to the period of the estrous cycle studied: proestrus and luteal phases. The lining epithelium was formed by four cellular strata in the proestrus phase: basale, spinosum, granulosum and corneum. Keratin was present in the lumen of the lateral vaginae in the proestrus phase. The stratum corneum and keratin were not observed in the luteal phase. These findings agree with the previous studies in the Grey short‐tailed opossum which is another Neotropical marsupial that inhabits Brazil and belongs to the Family Didelphidae. Moreover, this description makes available data that is fundamental to the studies of Neotropical marsupials reproductive biology.

## 1 Introduction

The white‐eared opossum ( *Didelphis albiventris* ) is a marsupial that belongs to the family Didelphidae and genus *Didelphis*. It is a species widely distributed in Brazil, occupying both open and forest formations and its distribution encompasses Brazilian biomes such as cerrado, caatinga and pampa, also occurring in the Atlantic Forest (Gardner 2008). It is an opportunistic species and tolerant to altered environments, being found in highly fragmented landscapes and even on the outskirts of urban centres (Melo and Sponchiado 2012; Rossi and Bianconi 2011).

Marsupials and eutherian mammals can be distinguished by a variety of characteristics, but it is their different modes of reproduction that most clearly lead to their classification (Behringer et al. 2006). The gestational period of marsupials is very short when compared to eutherian mammals of equivalent size. The gestation period in the genus *Didelphis* is 13 days and, in the genus *Monodelphis*, it is 15 days (Harder et al. 1993). The newborns are in a practically embryonic phase and attach themselves to the mother's teats located in the marsupium. In eutherian mammals, the time dedicated by the mother to gestation is approximately or exceeds the time between birth and weaning (Glazier 1990). In other words, in marsupials, the gestation period is very short, and the lactation period is very long (Thompson and Nicoll 1986).

Sepúlveda‐Vásquez et al. (2025) studied the reproductive tract and pouch anatomical variability across the reproductive phases (interestrus, proliferative and diestrus or luteal) in female *Didelphis marsupialis* . Tyndale‐Biscoe (2005) cited the proestrus, estrous and luteal phases. In the proestrus phase, mucus is secreted by the epithelial cells of the oviduct; there is hyperplasia of the uterine glands and epithelium, and hypertrophy of the vaginal complex with an increase in the thickness of the epithelium of this structure. This reaches its maximum in estrus when the lateral vagina increases several times.

The anatomy of the marsupials' female reproductive tract is unusual in some features when compared with that of eutherian animals. The anatomy of the marsupial female genital tract consists of two ovaries, oviducts, a double uterus, two lateral vaginae, a vaginal sinus and a urogenital sinus (Kress et al. 2004; Regli and Kress 2002; Schimming et al. 2018; Wick and Kress 2002). The two lateral vaginae, vaginal sinus and urogenital sinus constitute what is called the vaginal complex (Kress et al. 2004; Regli and Kress 2002; Schimming et al. 2018). The vaginal complex is the most variable part of the anatomy of the marsupial female tract (Regli and Kress 2002).

The vaginal complex of South American marsupials such as *Monodelphis domestica* has been studied by light, transmission and scanning electron microscopy (Kress et al. 2004; Regli and Kress 2002). A few years ago, the vaginal complex of the white‐eared opossum was described using gross anatomy and light microscopy by our research group (Schimming et al. 2018) and according to the authors' knowledge there is no ultrastructural study of the vaginal complex in this Neotropical marsupial, what motivated the development of this study. Thus, this study aimed to investigate the ultrastructure of the vaginal complex in the proestrus and metestrus phases of the female estrous cycle in the *D. albiventris* .

## 2 Material and Methods

### 2.1 Animals

Five female white‐eared opossums ( *D. albiventris* ) were used in this study. They were adults, sexually mature and weighed between 400 and 800 g and none of them were pregnant. The animals were from the Center for Medicine and Research in Wild Animals (CEMPAS), School of Veterinary Medicine and Animal Science, UNESP, Botucatu, São Paulo, Brazil. The marsupials were euthanised by an overdose of xylazine (1.5 mg/kg) and ketamine hydrochloride (20 mg/kg). The reproductive tracts were immediately removed, and the vaginal complex was processed either for transmission or scanning electron microscopy. This study was authorised by the Committee on the Use of Animals of the School of Veterinary Medicine and Animals Science, UNESP (CEUA 626/2014).

### 2.2 Reproductive Status

To determine the stage of the estrous cycle, the females were anaesthetised by intramuscular infusion of ketamine hydrochloride (20 mg/kg) and xylazine (1.5 mg/kg) before euthanasia, and vaginal smears were collected using a sterile cotton swab moistened with saline solution which was introduced into the female urogenital canal, with a slight rotation. The material in the swab was rubbed onto a clean histological slide and identified with the animal number. It was followed by fixation with alcohol‐ether mixture (1:1 ratio) and staining with haematoxylin and eosin (H&E). Three females were in proestrus and two in the luteal phase. No females in estrus or postestrus were available. It is worth noting that euthanasia was performed at CEMPAS due to traumatic causes and the impossibility of survival of these marsupials; therefore, it is not related to this study.

### 2.3 Transmission Electron Microscopy (TEM)

For TEM, tissues of vaginal complex (vaginal sinus and cranial, middle and caudal portions of the lateral vagina) were fixed by immersion in 2.5% glutaraldehyde and 4% paraformaldehyde solution in 0.1 M phosphate buffer (pH 7.3) for 24 h at room temperature and then postfixed in 1% osmium tetroxide in the same buffer for 2 h. After washing in distilled water, the samples were contrasted with an aqueous solution of 0.5% uranyl acetate for 2 h at room temperature, dehydrated in a graded acetone series and embedded in Araldite resin. Ultrathin sections were contrasted with uranyl acetate and lead citrate and then analysed and photographed with a Tecnai Spirit transmission electron microscope (FEI Company, Eindhoven, The Netherlands) at the Electron Microscopy Center of the Institute of Biosciences of Botucatu, UNESP.

### 2.4 Scanning Electron Microscopy (SEM)

One of the vaginal complex samples was isolated and fixed in 2.5% glutaraldehyde in 0.1 M phosphate buffer (pH 7.3) for 48 h at room temperature. Thereafter, the samples were washed in distilled water, postfixed in 1% osmium tetroxide diluted in distilled water for 30 min at room temperature, dehydrated through a graded series of ethanol, critical point‐dried with CO_2_ and coated with gold using a SCD 050 (Bal‐Tec, Los Angeles, CA, USA). The samples were examined and photographed using an FEI Quanta 200 Scanning Electron Microscope (FEI Company, Eindhoven, The Netherlands) with an accelerating voltage of 15 kV at the Electron Microscopy Center of the Institute of Biosciences of Botucatu, UNESP.

## 3 Results

**FIGURE 1:**
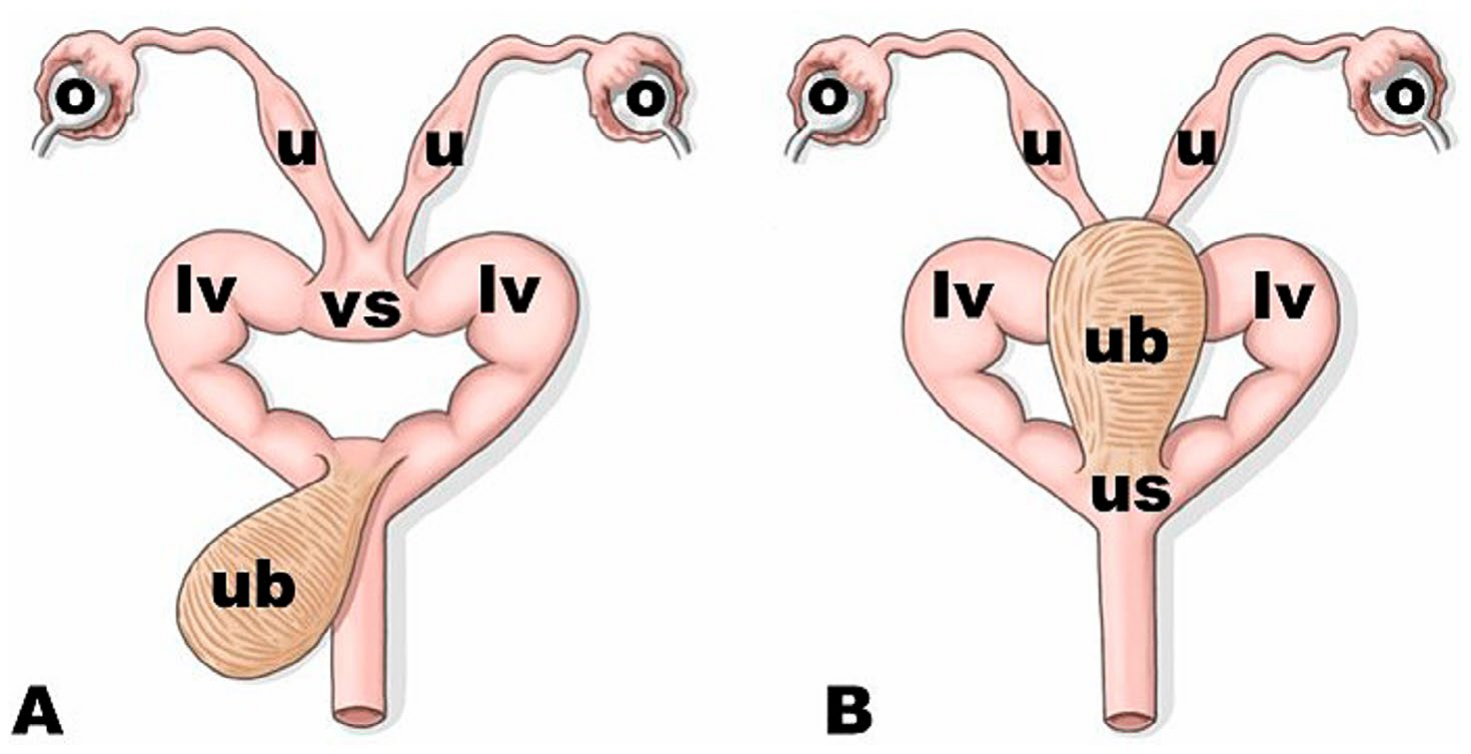
Schematic drawing representing the ventral view of the internal female genitalia found in the white‐eared opossum showing lateral vagina (lv), vaginal sinus (vs), uterus (u), ovarius (o), urogenital sinus (us) and urogenital canal (uc). In (A) the urinary bladder (ub) is displaced and in (B) it is the normal position.

The white‐eared opossum vaginal complex consisted of two lateral vaginae, a vaginal sinus and a urogenital sinus. The vaginal complex was related dorsally to the rectum and ventrally to the urinary bladder. Each lateral vagina occupied a diagonal position in relation to the vaginal and urogenital sinuses. Anatomically, the vaginal sinus was related cranially to the uterus and laterally to the lateral vaginas, which continued as two parallel canals to the urogenital sinus (Figure 1).

**FIGURE 2:**
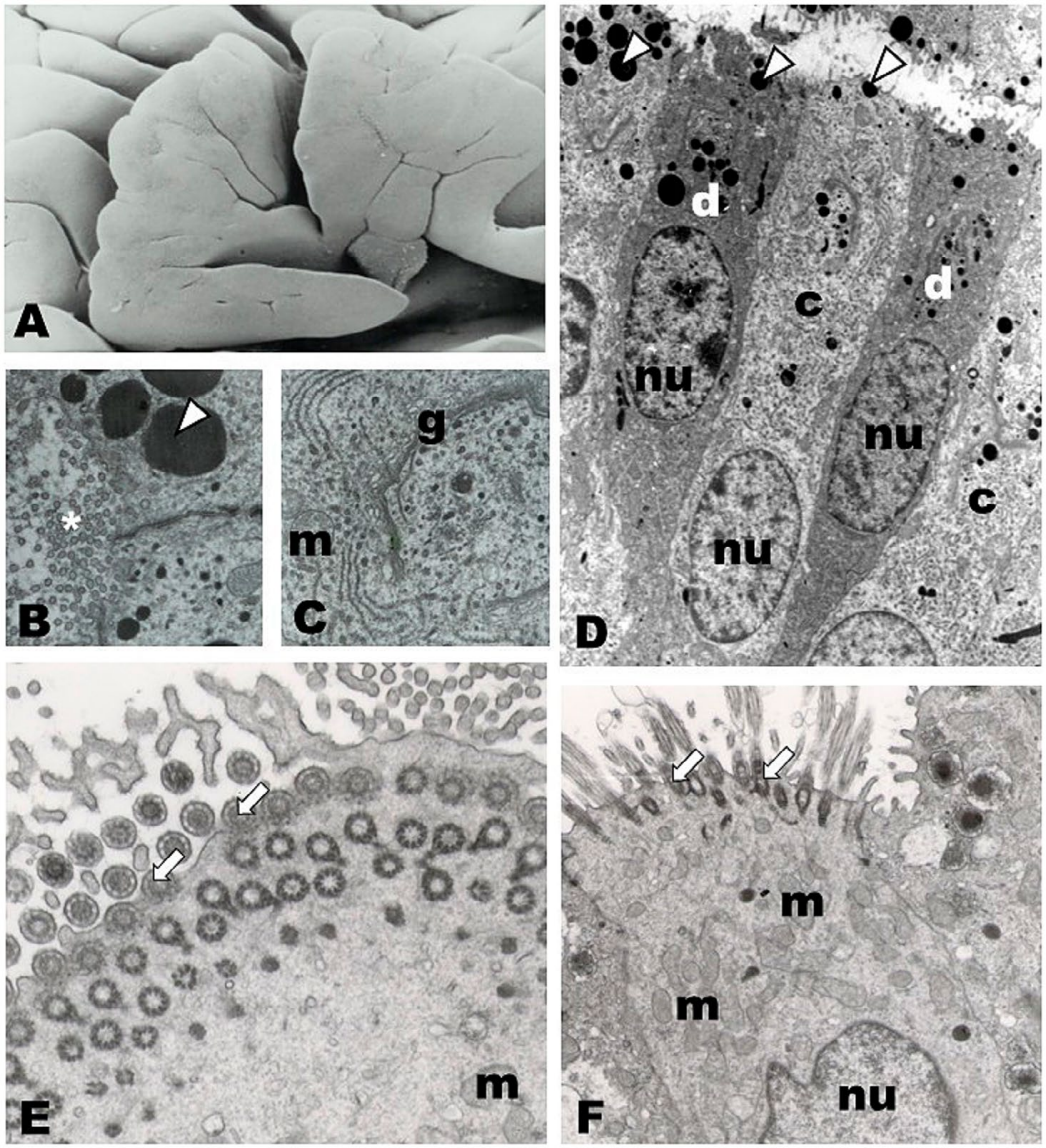
Scanning (A) and transmission (B–F) electron micrographs of the white‐eared opossum vaginal sinus. (A) General view of the vaginal mucosa arranged in longitudinal folds separated by deep furrows. ×206. Three cells types were observed in the epithelium: Clear (c), dark (d) and ciliated cells. (B, C) Clear cells showing mitochondria (m), Golgi complex (g), electron dense granules (white arrowheads) and microvilli (asterisk). B ×13,500, C ×14,400. (D) Note the nuclei (nu) and electron dense granules (white arrowheads) in the clear (c) and dark (d) cells. ×4800. (E, F) Mitochrondia (m), nuclei (nu) and cilia (white arrows) in the apical surface of the ciliated cells. E ×7875, F ×6620.

Ultrastructurally, the vaginal surface is thrown into numerous longitudinal folds separated by furrows (Figure 2A). Three cell types have been identified in the vaginal sinus epithelium: clear, dark and ciliated cells (Figure 2). The most abundant cells were clear cells, which were columnar cells that presented microvilli. These cells had rounded nuclei located in a basal position. The apical cytoplasm contained secretory granules. These granules showed a heterogeneous electron‐dense content (Figure 2B,D). The sarcoplasmic reticulum was abundant, and the Golgi complex and mitochondria were also observed (Figure 2C). Dark cells were characterised by having electron‐dense cytoplasm, irregularly shaped nuclei and many vacuoles. The apical cytoplasm of these cells had phagocytic and secretory cell characteristics (Figure 2B). Both cell types presented a large number of junctional complexes. Ciliated cells were rarely observed in the vaginal sinus epithelium and were characterised by the presence of cilia. They had cytoplasm devoid of granules, many small mitochondria, scarce sarcoplasmic reticulum and Golgi complex (Figure 2E,F).

**FIGURE 3:**
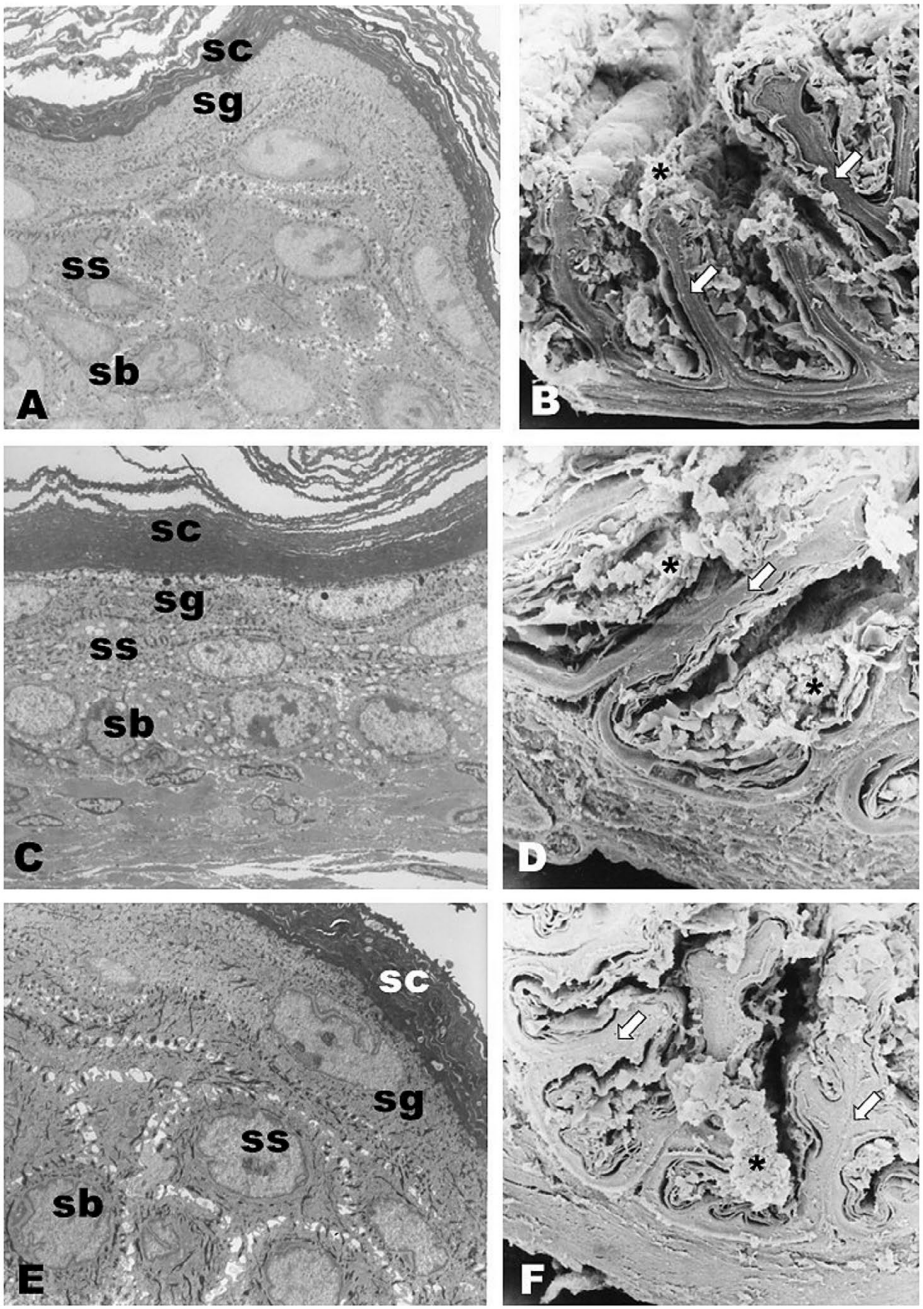
Transmission (A, C, E) and scanning (B, D, F) electron micrographs of the vaginal epithelium during the proestrus phase in the white‐eared opossum. (A, B) Cranial portion of the lateral vaginae. A ×2240, B ×107. (C, D) Middle portion of the lateral vaginae. C ×1860, D ×107. (E, F) Caudal portion of the lateral vaginae. E ×7300, F ×107. Note the four strata forming the vagina epithelium in the proestrus: Stratum corneum (sc), stratum granulosum (sg), stratum spinosum (ss) and stratum basale (sb). Scanning electron micrographs (B, D, F) indicate the mucosal folds (arrows) separated by deep furrows and the large amount of amorphous material (asterisks) in the cellular lumen.

**FIGURE 4:**
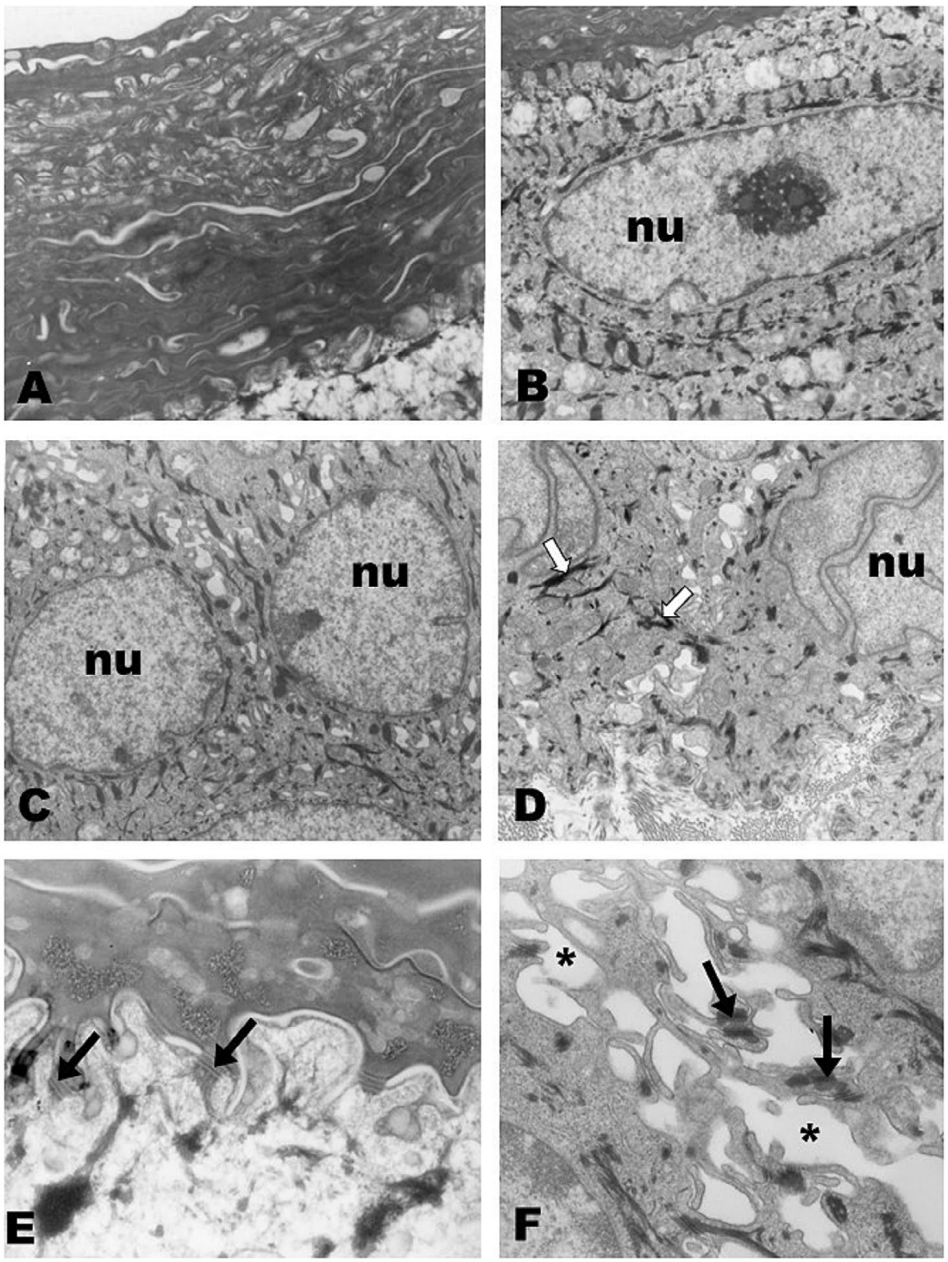
Transmission electron micrographs of details of the four strata of the vaginal epithelium during the proestrus phase in the white‐eared opossum. (A) Luminal layers of the stratum corneum. ×8600. (B) Nucleus (nu) of the cell in the stratum granulosum. ×7200. (C) Nuclei (nu) of the cells in the stratum spinosum. ×6700. (D) Nucleus (nu) of the cell in the stratum basale. White arrows indicate keratin filaments. ×9100. (E) Black arrows indicate cytoplasmic processes between stratum corneum and stratum granulosum. ×40,000. (F) Asterisks indicate distinct interdigitations and predominantly distended intercellular spaces between spinosum and basale strata. Arrows indicate junctional complex. ×23,000.

The lining epithelium of the lateral vaginae presented morphological changes according to the period of the estrous cycle studied: proestrus and luteal phases. The same morphological architecture was observed in the lining epithelium of the cranial, middle and caudal portions of the lateral vaginae (Figure 3). The lining epithelium was formed by four cellular strata in the proestrus phase: stratum basale (with small cubic cells), stratum spinosum (with 2 to 3 layers of polygonal cells), stratum granulosum (flattened cells and condensed nuclei) and stratum corneum (Figure 3A,C,E). These cellular strata, when observed through cross‐section under scanning electron microscopy, demonstrate the presence of amorphous material (keratin) present in the lumen of the lateral vaginae (Figure 3B,D,E). Through transmission electron microscopy, details of these layers could be observed (Figure 4). In general, the shape of the nucleus adapted to the shape of the cell, varying from oval to rounded (Figure 4B–D). The intercellular spaces were frequently distended, and the most notable morphological feature was the large number of junctional complexes between all strata cells (Figure 4E,F). The nuclei of the cells in the stratum basale were prominent and euchromatic. Small and few mitochondria, Golgi complex and rough endoplasmic reticulum were observed in moderate quantities in these cells. Keratin filaments appeared in thick bundles (Figure 4A,D). The cells of the stratum spinosum were polygonal in shape. The nucleus was heterochromatic, and the nuclear envelope was irregular; keratin filaments were also present in the cytoplasm. The cells of the stratum granulosum became elongated or flattened, and the intercellular spaces were very narrow. The nucleus was mostly heterochromatic with an obvious nucleolus (Figure 4B,C). The stratum corneum consisted primarily of concentric layers of keratin (Figure 4A). ‘Gutter’‐like processes connected the keratin layer to the cells of the stratum granulosum (Figure 4E).

**FIGURE 5:**
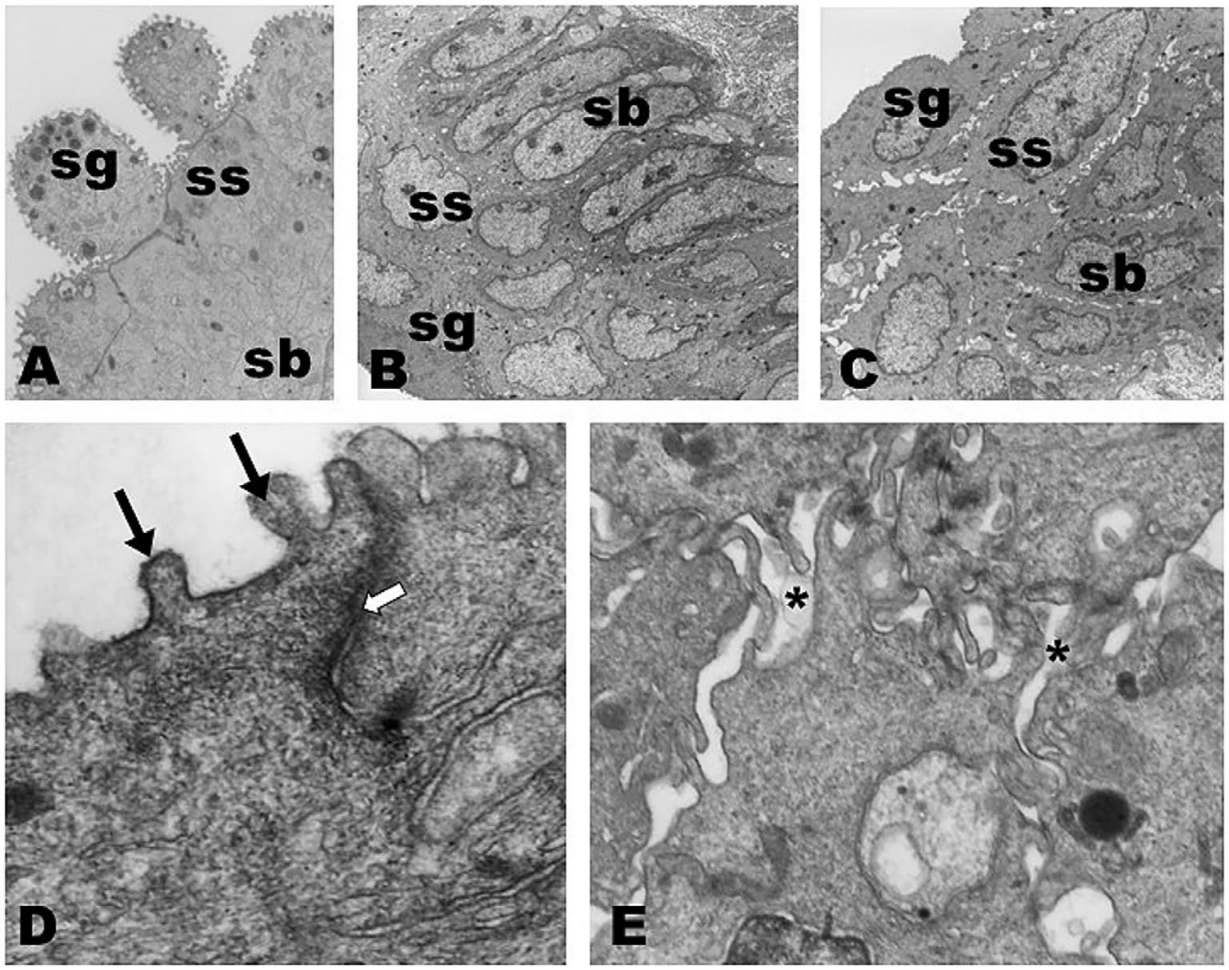
Transmission electron micrographs of the vaginal epithelium during the metestrus phase of the estrous cycle in the white‐eared opossum. Three strata forming the vaginal epithelium in the metestrus phase: Stratum granulosum (sg), stratum spinosum (ss) and stratum basale (sb). (A) Cranial portion of the lateral vaginae. ×3200. (B) Middle portion of the lateral vaginae. ×2200. (C) Caudal portion of the lateral vaginae. ×2800. (D) Black arrows indicate cellular microvilli of the stratum granulosum and white arrows junctional complex. ×23,000. (E) Asterisks indicate distended intercellular spaces between the strata. ×15,000.

Three cellular strata were observed in the lining epithelium of the lateral vagina in the luteal phase: stratum basale, stratum spinosum and stratum granulosum (Figure 5). The absence of the stratum corneum in the three portions of the lateral vagina was evident. The cells of the stratum granulosum had short, thick microvilli on their surface. Although the presence of three strata was observed in the three parts of the lateral vagina in the luteal phase of the white‐eared opossum, the morphology of the cells of the stratum granulosum varied according to the portion of the lateral vagina studied. In the cranial portion of the lateral vagina, they were prominent, as if they were coming out into the light (Figure 5A). In the middle portion of the lateral vagina, most cells were elongated, ranging from flattened to prominent, while in the caudal portion all the cells were flattened with variations only in size (Figure 5B,C). The morphological variation of the epithelial cells was only observed in the stratum granulosum, since the epithelial cells of the other strata (spinosum and basal) did not change their morphology independently of the parts of the lateral vagina. The cells of the stratum spinosum were mostly polygonal with rounded nuclei, while the cells of the stratum basale were extremely elongated with nuclei adapted to this shape. Many junctional complexes between cells of all layers and the distended intercellular space were ultrastructural features observed in the epithelium of the three portions of the lateral vaginae (Figure 5D,E).

## 4 Discussion

The present study described the ultrastructural features in the proestrus and luteal phases of the estrous cycle in the white‐eared opossum ( *D. albiventris* ). No females in estrus or diestrus phases were available in this study. The white‐eared opossum shows a seasonally polyestrous cycle and individual variations can occur mainly related to the amount of rainfall (Cerqueira 1984; Tyndale‐Biscoe 2005). Recent anatomical assessments of female *Didelphis marsupialis* by Sepúlveda‐Vásquez et al. (2025) demonstrate clear variability in the pouch and reproductive tract across the interestrus, proliferative and diestrus (luteal) phases, complementing the broader endocrinological stages (proestrus, estrous and luteal) previously detailed by Tyndale‐Biscoe (2005). The phase of the estrous cycle found in marsupials can be determined through vaginal smears, appearance of cornified epithelium in the urine or through histological sections of the lateral vagina. Regli and Kress (2002) identified the estrous cycle phases in the *Monodelphis domestica* using vaginal histology by either paraffin or semithin sections since in the case of vaginal smears, cells could derive from the sinus urogenitalis (Jurgelski and Porter 1974). In this study, the estrous cycle phases were determined using vaginal smears because it is a simple, economical and very efficient method.

Although there are several differences associated with the female reproductive tract of marsupials when compared to eutherians, the vaginal complex is the anatomical part that differs the most. The white‐eared opossum females possess a vaginal complex that comprises two lateral vaginae, a vaginal sinus and a urogenital sinus (Schimming et al. 2018).

The lateral vaginae found in marsupials can be compared to the vagina in eutherian mammals. However, unlike in eutherians where this structure has a direct link to the uterus, marsupials do not have a direct connection between the urogenital canal and the uteri, making the lateral vaginas the sole viable route for sperm transport in both non‐parous and most parous females (Tyndale‐Biscoe and Rodger 1978). The vaginal epithelium is a system that is constantly renewed and sensitive to oestrogen. The generative compartment is a homogeneous population of actively proliferating cells. The same basal cells can give rise to both a keratinised stratified squamous epithelium and a non‐keratinised epithelium (Regli and Kress 2002).

In relation to proestrus and luteal phases, the main difference is that in proestrus the epithelium is keratinised and in the luteal phase there is a loss of this keratinisation. These results agree with the results observed in the vaginal complex of other marsupials such as *Monodelphis domestica* (Kress et al. 2004; Regli and Kress 2002). The previous studies in *M. domestica* described the morphological characteristics of the vaginal complex in the estrous cycle including the presence of keratin and the number of epithelial layers in the vaginal complex epithelium depending on the estrous cycle phase (Kress et al. 2004; Regli and Kress 2002). Keratinisation begins at the end of proestrus, reaching maximum in estrus and would have a function attributable to protection against friction during copulation (Kress et al. 2004; Regli and Kress 2002). This keratinisation could be observed in the vaginal complex epithelium of the white‐eared opossum in the proestrus phase. This alternation between a keratinised and a nonkeratinised epithelium occurs under the influence of oestrogen and progesterone (Regli and Kress 2002).

In the white‐eared opossum, four cellular strata are observed in the proestrus phase: basale, spinosum, granulosum and corneum, being the cells of the stratum corneum covering the epithelium completely. These findings agree with the results found in the late proestrus of *Monodelphis domestica* since during met‐ to proestrus the epithelial keratinisation is promoted and the luminal metestrus cells are replaced by flattened cells of the stratum corneum (Regli and Kress 2002).

The lateral vagina increased in size and distended with mucus during the proestrus phase in the *Marmosa robinsoni* . Mucus‐secreting cells are in the cervix and on the inner surface of the uterine papilla, and no clear association has been found between the intensity of cornification or the amount of mucus and mating success (Godfrey 1975). Both the lateral vagina and the vaginal sinus of marsupials produce mucus during estrus and postestrus phases (Hughes and Rodger 1971; Rodger 1991). Risman (1947) suggested that the uterus of *Didelphis virginiana* should also produce mucus, although this has not been confirmed histologically.

Several functions have been attributed to the vaginal mucus of marsupials. Tyndale‐Biscoe and Renfree (1987) suggested that it takes part in the formation of the vaginal ‘plug’ by mixing with the semen. While Morgan (1946) suggests that, in the *Didelphis virginiana* , it has four functions: lubrication during copulation; sperm transport; aid in the passage of the fetus during birth; and, according to the author, it would have a mucilage function (demulcent) that would soften the mucous membrane. In the *Didelphis virginiana* , the transport of semen from the urogenital sinus to the cervix appears to occur due to a muscular action of the lateral vagina, although a plug was observed in the urogenital sinus and/or lateral vagina in didelphid marsupials (McCrady 1938).

In conclusion, the structural descriptions constitute an important substrate for other studies on the female reproductive system of *D. albiventris* and we conclude by suggesting that, while the human vaginal epithelium is non‐keratinised and protects itself against infection by reducing the pH, converting glycogen into lactic acid and secreting Ig (Kutteh and Mestecky 1994), in *Didelphis* as well as in *Monodelphis* (Regli and Kress 2002), since there is no glycogen deposit in the cells of the lateral vaginae, the pH remains neutral due to the production of mucus and subsequent keratinisation. Moreover, this description makes available data that is fundamental to the studies of Neotropical marsupials' reproductive biology.

## Conflicts of Interest

The authors declare no conflicts of interest.

## Data Availability

The data that support the findings of this study are available on request from the corresponding author. The data are not publicly available due to privacy or ethical restrictions.
